# Predictive factors for prolonged nutritional support after oesophagogastric cancer resection

**DOI:** 10.1308/rcsann.2025.0060

**Published:** 2026-01-20

**Authors:** P Chana, JL Moore, J Esteves-Cores, M Renna, J Lagergren, AR Davies, JA Gossage

**Affiliations:** ^1^ Imperial College London, UK; ^2^ Guy’s & St Thomas’ NHS Foundation Trust, UK; ^3^ King’s College London, UK; ^4^ Karolinska Institutet, Sweden

**Keywords:** Nutritional, Oesophagogastric, OR, Perioperative, Surgery

## Abstract

**Introduction:**

There remains great variation in the use of perioperative feeding adjuncts following oesophagogastric cancer resections with unknown clinical benefit. The aim of this study was to examine which preoperative clinicopathological factors were associated with prolonged use of adjuvant nutritional support after oesophagogastric cancer surgery and to evaluate the associated costs.

**Methods:**

A cohort study of 518 patients undergoing oesophagogastric resection and receiving perioperative parenteral nutrition was undertaken. Preoperative clinicopathological characteristics were evaluated using multivariable logistic regression, providing odds ratios (OR) with 95% confidence intervals (CI) and predictive factors for prolonged parenteral nutrition compared using receiver operator characteristic (ROC) analysis. An economic model was developed using complication rates related to parenteral nutrition and 2021 UK National Health Service tariffs.

**Results:**

Predictive factors for prolonged parenteral nutrition use included: age >65 vs ≤65 years (OR 1.83, 95% CI 1.22–2.76), >10% preoperative weight loss (OR 2.20, 95% CI 1.03–4.70), open vs minimally invasive surgery (OR 1.64, 95% CI 1.03–2.62) and neck vs abdominal anastomosis (OR 2.54, 95% CI 1.35–4.79). ROC analysis provided an area under the curve of 0.72. The projected annual unit savings were £75,912 if parenteral nutrition was reserved for high-risk patients.

**Conclusions:**

This study identified factors associated with prolonged nutritional support after oesophagogastric surgery. As practice evolves towards minimally invasive surgery and enhanced recovery protocols with low complication rates, short-course adjuvant feeding may not be necessary for patients who progress promptly to appropriate oral intake. A tailored treatment pathway, excluding routine use of perioperative feeding adjuncts for low-risk patients may lead to considerable cost savings.

## Introduction

In the era of evidence-based practice, there remains much variation in perioperative nutritional strategies for patients undergoing oesophagogastric cancer resection.^1^ Nutritional deficiencies are common in these patients, and during preoperative assessments and neoadjuvant therapy, dysphagia and weight loss often progress. Traditionally it was not unusual to encounter malnourished and frail patients at the time of resection, contributing to adverse postoperative outcomes.^2,3^ The use of feeding adjuncts in the pre- and perioperative settings is therefore well recognised in oesophagogastric surgery, but there is no consensus as to which method is most effective. Surgically placed feeding jejunostomy tubes are the mainstay of feeding adjuncts and are inserted routinely in 56% of units in the United Kingdom (UK), with nasojejunal feeding, parenteral nutrition and oesophageal stenting as alternative options.^4,5^

However, oesophagogastric cancer surgery has evolved over the past two decades. Improvements in staging and fitness assessments have resulted in stricter selection criteria for determining potential surgical candidates, and increased use of neoadjuvant therapies often improves patients' oral nutrition. The addition of preoperative prehabilitation can also optimise patients' physiology, fitness and diet.^6^ In the UK, surgery has been centralised to high-volume units with appropriate specialisms, including dietetics, and the introduction of minimally invasive surgery has facilitated the implementation of enhanced recovery pathways that promote early postoperative oral nutritional intake.^7^

Despite this, adjuvant feeding support is often used in the perioperative setting to mitigate complications, including anastomotic leak. Although traditionally a significant problem following oesophagogastric resection, with modern surgical techniques and patient optimisation, clinically significant anastomotic leak rates are now observed in approximately 10% of patients.^8^ Therefore, the need for routine adjuvant feeding may be negated as more appropriately selected patients overcome surgery without serious complications or postoperative malnutrition, and insertion of feeding tubes as an ‘insurance policy’ is not required.^9^

Furthermore, complications associated with feeding tubes and lines are a significant cause of potentially avoidable perioperative morbidity given that they often go unused, and their benefit remains unproven.^10^ There are also economic costs associated with feeding adjuncts, including time and expertise taken for insertion, dose calculation and delivery of the appropriate feeding regimen, equipment/materials, patient education and management of complications.^11^

We hypothesised that routine use of adjuvant feeding is unnecessary for patients who progress well through the postoperative pathway and meet oral nutritional goals early, and that supplementary feeding should be reserved only for patients with an increased risk of requiring prolonged nutritional support.

The primary aim of this study was to identify preoperative clinicopathological factors associated with prolonged parental nutrition use after oesophagogastric surgery. Other aims were to evaluate complications and costs associated with adjuvant parenteral nutrition use, and to develop a risk model to determine which patients benefit from postoperative nutritional support.

## Methods

### Setting

Guy's and St Thomas' NHS Foundation Trust hosts a high-volume oesophagogastric cancer unit in London, UK, which over the past ten years has transitioned to a minimally invasive practice with favourable postoperative outcomes given the volume and complexity of case-mix seen, (trans-hiatal and three-stage oesophagectomies, salvage resections and complex reconstructions are also regularly performed).^4^

Since 2015, as part of the unit's enhanced recovery pathway, all patients undergoing oesophagogastric resection receive supplementary parenteral nutrition in the postoperative period. Patients are kept on minimal oral intake until a water-soluble contrast swallow is performed. Without evidence of hold up or anastomotic leak, oral intake is progressed, and parenteral nutrition is weaned down with shared decision making between surgeons, perioperative physicians and dieticians regarding nutrition on a case-by-case basis. As with many oesophagogastric cancer units, patients follow the same enhanced recovery pathway regardless of operative approach and jejunostomy tubes are not routinely used.

### Design

This was a cohort study based on an ethically approved, prospectively maintained database of patients who underwent surgery with curative intent for oesophageal or gastric cancer at Guy's and St Thomas' NHS Foundation Trust between 2016 and 2023.

Results of staging investigations were reviewed by a specialist multidisciplinary team and neoadjuvant treatment was considered for patients with tumours staged beyond cT1 cN0. Patients undergoing open or minimally invasive oesophagectomy or total gastrectomy for all histological subtypes were included.

Perioperative nutrition data were collected following a thorough review of electronic patient records.

The primary outcome was prolonged use of postoperative parenteral nutrition, which was defined as 9 days or more during the index admission. The decision to define prolonged use as 9 days or more was based upon an initial data review that demonstrated a median duration of parenteral nutrition use of 8 days; hence an above median cutoff was chosen. Secondary outcomes were complications and costs related to use of postoperative parenteral nutrition.

### Postoperative nutrition

All patients received the standard postoperative feeding protocol of parenteral nutrition delivered via a centrally placed venous catheter, commencing on postoperative day 1. Oral intake was initiated providing a contrast swallow performed on day 3 demonstrated no contraindications. Parenteral nutrition was discontinued once a patient was established on sufficient oral intake following consultation with a specialist oesophagogastric dietician. Patients unable to commence oral feeding or with nutritional concerns remained on parenteral nutrition as inpatients or had enteral feeding tubes placed to facilitate discharge. These patients remained supported by dieticians until they were able to meet their nutritional requirements orally.

### Statistical analysis

Clinicopathological characteristics were compared using the chi-squared test. Associations between these characteristics and risk of prolonged parenteral nutrition use were evaluated using logistic regression analysis, providing odds ratios (OR) with 95% confidence intervals (CI). A crude model did not adjust for any covariates. A multivariable model adjusted for: age (≤65 or >65 years) in keeping with the established literature, sex (male or female), comorbidity (Charlson Comorbidity Index score 0–2 or >2), tumour histology (adenocarcinoma, squamous cell carcinoma or other), cT stage (cT0–2 or cT3–4), cN stage (cN0 or cN+), neoadjuvant treatment (none or chemotherapy or chemoradiotherapy), anastomosis location (abdomen, chest or neck, in keeping with the variety of operative techniques routinely used at Guy's and St Thomas'), surgical access (open or minimally invasive), body mass index (BMI; <18, 18–25 or >25), preoperative weight loss (none, 0%–10%, >10% or not recorded), and preoperative supplementary feeding with either a feeding tube or parenteral nutrition (yes or no).

The ability of clinicopathological characteristics to predict prolonged parenteral nutrition use were compared using receiver operator characteristic (ROC) analysis to calculate the area under the curve (AUC). An AUC of 1.0 indicated perfect predictive ability and an AUC of 0.5 indicated no ability. Values of *p* <0.05 were deemed statistically significant. Statistical analysis was performed using IBM SPSS statistics (IBM Corp, IBM SPSS statistics version 27.0. Armonk, NY, USA).

### Cost analysis

Following oesophagogastric resection, patients were modelled to receive a 9-day course of parenteral nutrition, and associated costs were calculated. Within the model, the costs of initiating parenteral nutrition, complications of receiving it and the associated costs of treating these were included and costed using the most recent UK National Health Service (NHS) tariffs, the British National Formulary, NHS best practice guidelines, local protocols and procedures alongside the Agenda for Change pay agreements.^12–14^ The total cost of parenteral nutrition provision included medication costs, time to prepare each solution by specialist pharmacists and a daily review by a qualified dietitian. The methodology for calculating complication rates was decided upon using recent systematic reviews and a single rate was found by taking the midpoint of ranges if given. The total costs for the treatment of each complication were then multiplied by the complication rates using unit data to give an accurate estimate of the pooled costs. Because parenteral nutrition was given to all postoperative patients, this analysis aimed to review the provision of adjuvant nutrition and categorise patients into normal (less than 9 days of postoperative parenteral nutrition) or prolonged use (9 or more days of postoperative parenteral nutrition). The proportion of such patients were multiplied by the average number of patients seen per year over the last three complete years (January to January) and the total costs of parenteral nutrition provision was included to give the estimated savings associated with not providing it to patients in the normal subgroup.

## Results

### Patients

The final cohort included 518 patients (Table 1). Median age was 66 years, and the majority were male (81.1%). Median duration of parenteral nutrition was 8 days. Median total length of stay was 10 days. Most patients underwent neoadjuvant chemotherapy (84.2%) and a minority received neoadjuvant chemoradiotherapy (2.3%). Most patients underwent oesophagectomy (84.2%) with the remainder undergoing total gastrectomy (15.8%). In total, 185 patients (35.7%) received prolonged parenteral nutrition after surgery (Table 2). Patients with prolonged parenteral nutrition were more likely to have an anastomosis located in the neck (41.1% vs 26.1%), have undergone open surgery (50.3% vs 38.7%), have had preoperative weight loss (53.5% vs 37.5%), and have suffered from a postoperative complication (64.9% vs 30.3%), including anastomotic leak (20.0% vs 2.1%) and pneumonia (33.0% vs 12.6%). Of the patients receiving prolonged parenteral nutrition, 54 (10% of the total cohort) required a feeding tube insertion (either nasojejunal or surgically placed jejunostomy) to supplement longer term feeding and facilitate discharge home.

**Table 1:** Patient characteristics stratified by normal (<8 days) or prolonged (**≥**9 days) postoperative parenteral nutrition (PN) after surgical resection for oesophagogastric cancer

Variable	PN ≤8 days (*n* = 333)	PN ≥9 days (*n* = 185)	*p*-value
Age at operation (median)	65 years	68 years	**0.002**
Sex			
Male	269 (80.8)	151 (81.6)	0.815
Female	64 (19.2)	34 (18.4)	
Charlson Comorbidity Index score			
0–2	310 (93.1)	164 (88.6)	0.082
>2	23 (6.9)	21 (11.4)	
Tumour histology			
Adenocarcinoma	312 (93.7)	168 (90.8)	0.470
Squamous cell carcinoma	18 (5.4)	15 (8.1)	
Other	3 (0.9)	2 (1.1)	
cT stage			
cT0–2	71 (21.3)	39 (21.1)	0.28
cT3–4	260 (78.1)	142 (76.8)	
Not recorded	2 (0.6)	4 (2.2)	
cN stage			
cN0	85 (25.5)	39 (21.1)	0.16
cN+	246 (73.9)	142 (76.8)	
Not recorded	2 (0.6)	4 (2.2)	
Operation			
Ivor-Lewis oesophagectomy	107 (32.1)	52 (28.1)	**0.002**
Left thoraco-abdominal oesophagectomy	78 (23.4)	33 (17.8)	
Trans-hiatal oesophagectomy	71 (21.3)	68 (36.8)	
Three-stage oesophagectomy	16 (4.8)	11 (5.9)	
Total gastrectomy	61 (18.3)	21 (11.4)	
Anastomosis location			
Abdomen	61 (18.3)	21 (11.4)	**0.001**
Chest	185 (55.6)	88 (47.6)	
Neck	87 (26.1)	76 (41.1)	
Surgical access			
Open	129 (38.7)	93 (50.3)	**0.011**
Minimally invasive	204 (61.3)	92 (49.7)	
Neo-adjuvant therapy			
None	55 (16.5)	29 (15.7)	0.952
Chemotherapy	270 (81.1)	152 (82.2)	
Chemoradiotherapy	8 (2.4)	4 (2.2)	
Body mass index			
<18	2 (0.6)	1 (0.5)	0.074
18–25	88 (26.4)	68 (36.8)	
>25	241 (72.4)	116 (62.7)	
Not recorded	2 (0.6)	0 (0)	
Preoperative weight loss			
None	166 (49.8)	69 (37.3)	**0.004**
0%–10%	107 (32.1)	80 (43.2)	
>10%	18 (5.4)	19 (10.3)	
Not recorded	42 (12.6)	17 (9.2)	
Preoperative supplementary feeding			
No	318 (95.5)	171 (92.4)	0.146
Yes	15 (4.5)	14 (7.6)	
Preoperative albumin (g/l)			
<34	7 (2.1)	6 (3.2)	0.633
>34	294 (88.3)	164 (88.6)	
Not recorded	32 (9.6)	15 (8.1)	
Surgical complications			
None	232 (69.7)	65 (35.1)	**<0.001**
Any	101 (30.3)	120 (64.9)	
Anastomotic leak			
No	326 (97.9)	148 (80.0)	**<0.001**
Yes	7 (2.1)	37 (20.0)	
Pneumonia			
No	291 (87.4)	124 (67.0)	**<0.001**
Yes	42 (12.6)	61 (33.0)	

Values are given as *n* (%) unless indicated otherwise.

**Table 2:** Total duration of postoperative parenteral nutrition in patients undergoing surgical resection for oesophagogastric cancer

Total duration of parenteral nutrition (days)	Number (%)
<7	114 (21.6)
7	131 (24.8)
8	88 (16.6)
9	72 (13.6)
10	32 (6.0)
>10	81 (15.3)

### Risk of need for prolonged parenteral nutrition

Preoperative predictive factors for prolonged parenteral nutrition use on adjusted logistic regression analysis (Table 3) included age >65 vs ≤65 years (OR 1.83, 95% CI 1.22–2.76), preoperative weight loss (0%–10% total weight loss: OR 1.65, 95% CI 1.08–2.54; >10% total weight loss: OR 2.20, 95% CI 1.03–4.70) compared with no weight loss, open vs minimally invasive surgery (OR 1.64, 95% CI 1.03–2.62) and an anastomosis located in the neck vs abdominal anastomosis (OR 2.54, 95% CI 1.35–4.79). Patients with a BMI over 25 had a lower likelihood of prolonged parental nutrition use compared with those with a BMI less than 25 (OR 0.63, 95% CI 0.42–0.96). Sex, Charlson Comorbidity Index score, neoadjuvant treatment regimen, chest anastomosis, tumour histology, clinical staging and preoperative supplementary feeding were not statistically significantly associated with prolonged parenteral nutrition. Subgroup analysis evaluating patients with oesophageal cancer undergoing oesophagectomy demonstrated similar results to the whole cohort.

**Table 3:** Odds ratios (OR) with 95% confidence intervals (CI) of prolonged parenteral nutrition use in patients (*n* = 518) undergoing surgical resection for oesophagogastric cancer

Variable	Crude	Adjusted
	OR (95% CI)	OR (95% CI)
Age		
≤65 years	1.00 (reference)	1.00 (reference)
>65 years	1.82 (1.26–2.62)	1.83 (1.22–2.76)
Sex		
Male	1.00 (reference)	1.00 (reference)
Female	0.95 (0.60–1.50)	1.01 (0.60–1.70)
Charlson comorbidity score		
0–2	1.00 (reference)	1.00 (reference)
>2	1.73 (0.93–3.21)	1.42 (0.73–3.03)
Histology		
Adenocarcinoma	1.00 (reference)	1.00 (reference)
Squamous cell carcinoma	1.55 (0.76–3.15)	1.43 (0.60–3.37)
Other	1.24 (0.21–7.48)	1.27 (0.15–10.50)
cT stage		
cT0–2	1.00 (reference)	1.00 (reference)
cT3–4	0.99 (0.64–1.55)	1.06 (0.58–1.95)
cN stage		
cN0	1.00 (reference)	1.00 (reference)
cN+	1.26 (0.82–1.94)	1.25 (0.73–2.15)
Neoadjuvant treatment		
None	1.00 (reference)	1.00 (reference)
Chemotherapy	1.07 (0.65–1.75)	1.48 (0.73–3.03)
Chemoradiotherapy	0.95 (0.26–3.42)	0.71 (0.15–3.32)
Anastomosis location		
Abdomen	1.00 (reference)	1.00 (reference)
Chest	1.34 (0.76–2.33)	1.72 (0.89–3.34)
Neck	2.64 (1.47–4.72)	2.54 (1.35–4.79)
Surgical access		
Minimally invasive	1.00 (reference)	1.00 (reference)
Open	1.59 (1.11–2.30)	1.64 (1.03–2.62)
Body mass index		
18–25	1.00 (reference)	1.00 (reference)
<18	1.65 (0.06–7.29)	1.61 (0.12–21.09)
>25	0.62 (0.42–0.92)	0.63 (0.42–0.96)
Weight loss		
None	1.00 (reference)	1.00 (reference)
0%–10%	1.80 (1.20–2.69)	1.65 (1.08–2.54)
>10%	2.54 (1.26–5.13)	2.20 (1.03–4.70)
Supplementary feeding		
No	1.00 (reference)	1.00 (reference)
Yes	1.74 (0.82–3.68)	1.13 (0.49–2.62)

### Prediction model for prolonged parenteral nutrition

**Figure 1:**
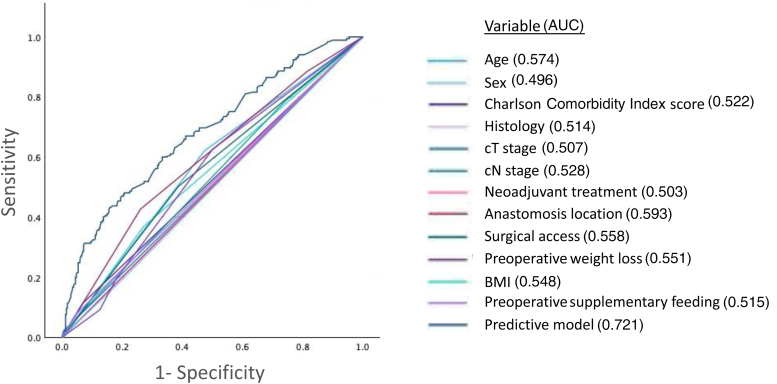
Receiver operator characteristic (ROC) analysis evaluating the ability of different clinicopathological characteristics to predict prolonged parenteral nutrition use following surgical resection for oesophagogastric cancer

ROC analysis (Figure 1) demonstrated that anastomosis location (AUC 0.59), age (AUC 0.57) and surgical access (AUC 0.56) were individually weak predictive factors for prolonged parenteral nutrition use; however, combination of the parameters in the multivariable model provided a higher AUC of 0.72. A model including only statistically significant characteristics (Table 4) (age >65, cervical anastomosis, open surgery, >10% weight loss) predicted prolonged parenteral nutrition use with a sensitivity of 52.4%, specificity of 67.3%, positive predictive value (PPV) of 47.1% and negative predictive value (NPV) of 71.8% if two or more risk factors were present.

**Table 4:** Prognostic ability of risk model to predict prolonged postoperative parenteral nutrition (PN) use after surgical resection for oesophagogastric cancer

	Parenteral nutrition use							
	PN ≥9 days	PN ≤8 days	AUC	Sensitivity	Specificity	PPV	NPV	*p*-value
≥1 risk factor								
High risk^*^	154 (38.0%)	251 (62.0%)	0.54	83.2%	24.6%	38.0%	72.6%	0.04
Low risk	31 (27.4%)	82 (72.6%)						
≥2 risk factors								
High risk^†^	97 (47.1%)	109 (52.9%)	0.60	52.4%	67.3%	47.1%	71.8%	**<0.001**
Low risk	88 (28.2%)	224 (71.8%)						
≥3 risk factors								
High risk^‡^	51 (63.0%)	30 (37.0%)	0.60	27.6%	91.0%	63.0%	69.4%	**<0.001**
Low risk	134 (30.7%)	303 (69.3%)						

AUC = area under the curve; NPV = negative predictive value; PPV = positive predictive value

*One or more risk factors (age >65, presence of neck anastomosis, >10% weight loss, open surgery)

†Two or more risk factors (age >65, presence of neck anastomosis, >10% weight loss, open surgery)

^‡^Three or more risk factors (age >65, presence of neck anastomosis, >10% weight loss, open surgery)

### Cost analysis

The cost of supplying a 9-day course of parenteral nutrition was modelled using an example 112 patients per year based on unit data (Table 5). This demonstrated a projected cost savings of £75,912 per annum by limiting the use of perioperative parenteral nutrition to patients that were predicted to need 9 or more days of parenteral nutrition (26.1% of resections).

**Table 5:** Cost of supplying a 9-day course of parenteral nutrition (PN) to patients undergoing oesophagogastric cancer resection

	Cost
Nine-day course of PN per patient:	£884
Associated complications per patient:	£176
Total cost of PN per patient:	£1,060
Proportion of low-risk patients:	63.9%
Patients seen per year:	112
Projected service savings per year:	£75,912

### Complications of parenteral nutrition

Parenteral nutrition and line-related complications (Table 6) included culture-confirmed line infections (*n* = 7, 1.4%) and line thrombus (*n* = 11, 2.1%). Line infections were treated with intravenous antibiotics and removal of the infected line. Line thrombus was treated with anticoagulation for 3 months. There were no patients with thrombo-embolism or other adverse clinical sequalae related to line infection or thrombus. In total 93 patients (18.0%) required insertion of a peripherally inserted central catheter for delivery of nutrition.

**Table 6:** Complications related to use of parenteral nutrition in patients undergoing surgical resection for oesophagogastric cancer

Complication	*n* (%)
Culture-confirmed line infection	7 (1.4)
Line thrombus	11 (2.1)
Thrombo-embolism secondary to line thrombus	0 (0.0)
Line blockage/displacement	17 (3.3)

## Discussion

In this study, several patient- and treatment-related factors were associated with requiring prolonged adjuvant nutritional support after oesophagogastric surgery. Risk factors included advanced age, significant preoperative weight loss, an open surgical approach and an anastomosis located in the neck, whereas being overweight reduced this risk.

There is a paucity of evidence evaluating perioperative nutrition in oesophagogastric cancer, including the use of parenteral nutrition. Some studies have shown that early enteral feeding is beneficial compared with parenteral nutrition; however, this is only in the context of patients not experiencing complications.^15^

Data on perioperative feeding adjuncts are varied and although calorific intake is maintained with the use of adjuncts, studies have not explored other nutritional outcomes such as loss of muscle mass or sarcopenia. There is emerging evidence that reducing sarcopenia during neoadjuvant treatment improves outcomes; however, this has not been replicated in the perioperative period and the benefits of adjunctive feeding are poorly understood, including its effect on patient quality of life and mental health.^16^

Despite being the most common feeding adjunct used following oesophagogastric surgery, surgically placed feeding jejunostomy tubes can cause significant morbidity including necrotising enterocolitis in patients with sepsis or on inotropic support, and tube-related problems such as torsion and bowel obstruction.^17^ Therefore, intravenous parenteral nutrition may be a safer, short-term alternative for patients requiring adjuvant nutritional support after surgery and this study has demonstrated that parenteral feeding delivery, via a central venous catheter (with an isolated lumen for feeding) routinely inserted during resection, is feasible and has a low risk profile. There was a low incidence of complications, including a low line infection and symptomatic upper limb thrombosis rate, likely in part related to improvements in line care following the universal adoption of the Matching Michigan Project.^18^ Utilisation of custom-made parenteral nutrition was also safe and there was no evidence of re-feeding syndrome. For patients requiring extended nutritional support during hospital admission, ongoing nutrition delivery via a peripherally inserted central catheter line was a safe and effective technique for preventing prolonged central venous catheter use.

Although parenteral feeding has been the preferred adjunct of choice in the host institution because of the well-established pathways of care, the use of other feeding adjuncts, such as nasojejunal feeding tubes and surgically placed feeding jejunostomy tubes, can be applied to this methodology to examine practice in other centres, and future work may examine differences in outcomes following the use of these adjuncts in the perioperative setting.

Perioperative complications were more commonly associated with patients receiving prolonged nutritional support. However, approximately one in three of these patients had no complications, and one in three patients who had standard duration parenteral nutrition did. This suggests that other factors (besides complication rates) were involved in determining the duration of adjuvant supplementary feeding for a substantial number of patients. Although some of the prognostic factors identified in this study for prolonged nutritional support were similar to recognised risk factors associated with perioperative complications following oesophagogastric resection (e.g. cervical anastomosis, age), there were some differences (e.g. patient comorbidity and high BMI, which were not associated with prolonged nutrition).^19–21^

### Study limitations

Some methodological issues deserve attention. This study allowed for the long-term follow-up of a large, consecutive cohort of patients undergoing oesophagogastric resection. Although data were collected prospectively, the observational design made it impossible to rule out confounding factors. As a single-centre study, one advantage was that all patients underwent the same perioperative treatment pathway with reduced heterogeneity. However, this design reduced the generalisability of the findings compared with a population-based or multicentre approach and external validation of these findings is required; this is an intended area of future work. Because enhanced recovery protocols are not standardised, different results may be obtained in different units. For example, in the enhanced recovery protocol utilised in this study, a water-soluble swallow was performed before the initiation of oral intake, typically on day 3; however, this is not the case at all institutions, which do not routinely use contrast imaging before commencing oral intake.

Patients undergoing both oesophagectomy and total gastrectomy were included in this study. Although this had the potential to introduce confounding, because patients with tumours of the gastro-oesophageal junction may undergo either operative approach and all patients follow the same postoperative feeding protocol, inclusion of both cohorts was felt to be a more accurate representation of current clinical practice in the UK. Furthermore, the optimum perioperative feeding strategy remains unknown for all patients undergoing oesophagogastric resectional surgery, and subgroup analysis revealed similar results regardless of operation performed.

## Conclusions

This study has identified factors associated with prolonged parenteral nutrition use after oesophagogastric resection and supports the development of tailored enhanced recovery protocols with focused implementation of adjuvant feeding regimes for patients with preoperative high-risk characteristics. This is currently being developed at the host institution with plans to commence a prospective trial evaluating whether avoiding routine adjuvant perioperative parenteral nutrition for ‘low-risk’ patients can be safely implemented in clinical practice. This group will instead commence on oral intake on postoperative day 3 following a satisfactory water-soluble contrast swallow. The predictive model in this study was able to predict parenteral nutrition usage of less than 9 days with reasonable accuracy for low-risk patients (approximately 70%).

The ability to identify high-risk patients in the preoperative setting will allow for targeted intensive dietician support for these vulnerable patients, which may improve clinical outcomes and quality of life. It is also hoped that low-risk patients who commence early oral intake, without the need for adjuvant nutritional support, will experience improved satisfaction during their perioperative journey. These findings will be recorded by members of the multidisciplinary team and used to inform future research projects.

As well as delivering good clinical care, modern healthcare providers also need to consider the economic cost of interventions and this study is an example of how clinical research can generate cost savings. The potential cost saving achieved by no longer routinely giving low-risk patients adjuvant parenteral nutrition can be re-invested within the unit to improve patient care in other domains. Dietician time and resources will also be freed to focus on high-risk patients and other important aspects of oesophagogastric cancer surgery such as ‘survivorship’ and the functional and nutritional problems commonly experienced by this group after discharge from hospital which cause significant morbidity.^22^ More targeted resources can also be directed to those patients who fail to progress to feeding without the need for adjuncts, as seen in 10% of this cohort. A national nutritional audit commencing in the UK aims to capture perioperative nutritional data to help inform practice and evaluate the availability of allied specialists such as dietetics to help with service planning, and will be a welcome addition to oesophagogastric cancer care in the UK.^23^

Multicentre randomised trials are urgently needed to determine whether adjuvant feeding is necessary for oesophagogastric patients, and if so, which feeding adjuncts are the most effective and safe. However, it is acknowledged that the required standardisation of the entire perioperative pathway for all participating centres may be challenging because of the significant variation in current practice observed between units, although this would be of great importance to reduce confounding in any future trial design.

It is important to recognise the strides that the global oesophagogastric community has taken over the past two decades to improve patient care. The specialty is now well established and delivers low perioperative mortality and morbidity rates, which have been further improved by recent advances such as centralisation, prehabilitation, minimally invasive surgery and enhanced recovery. Despite this, oesophagogastric multidisciplinary teams need to continue to improve outcomes and perioperative nutrition is an essential component of this.

In conclusion, this study identified risk characteristics for prolonged need for nutritional support following oesophagogastric cancer resection that should be considered in the preoperative setting with development of tailored treatment pathways. Many low-risk patients can potentially avoid routine feeding adjunct use, reducing perioperative morbidity and healthcare costs.

## Data availability

Data supporting the findings of this study are available in the paper and the online supplementary material.
